# Cochlear Implant Complicated by Nontuberculous Mycobacteria Infection: Report and Literature Review

**DOI:** 10.1155/crot/5973005

**Published:** 2025-04-30

**Authors:** Haidee Chen, Erik B. Vanstrum, Rodell Santuray, Adam Xiao, Akira Ishiyama

**Affiliations:** ^1^David Geffen School of Medicine, University of California, Los Angeles, California, USA; ^2^Department of Head and Neck Surgery, University of California, Los Angeles, California, USA

**Keywords:** cochlear implant, infection, nontuberculosis mycobacterium

## Abstract

**Objective:** Nontuberculosis mycobacteria (NTM) infection of cochlear implants are exceedingly rare. Here, we report one such case and review the literature surrounding previous reports.

**Methods:** Case report.

**Case Report:** A 76-year-old female underwent right cochlear implantation. Her course was complicated by wound dehiscence, three surgical debridements, and ultimately explantation. Cultures ultimately grew *Mycobacterium abscessus*, which was effectively treated with Azithromycin, Omadacycline, and 3 months of injectable Cefoxitin. At the latest follow-up, the patient is without evidence of further infection and pending reimplantation.

**Conclusion:** NTM is a rare cause of postsurgical infections following cochlear implantation. It is especially important to consider this in cases of indolent, antibiotic-resistant infections to optimize patient treatment.

## 1. Introduction

While cochlear implantation (CI) infections are rare, with reports indicating rates between 1% and 8%, the complication is serious, resulting in significant morbidity and mortality [1–4]. Treatment frequently involves aggressive culture-driven antibiotic regimens, surgical debridement, and in some cases, explantation [2, 4, 5].

The most commonly identified pathogens include *Staphylococcus aureus, Pseudomonas aeruginosa,* and other normal skin flora [5, 6]. However, rare pathogens have been documented as causative agents, including nontuberculosis mycobacteria (NTM) [7, 8]. Identification and management of such organisms is challenging and necessitates prompt, aggressive treatment [7, 8]. As the incidence of NTM infections is on the rise globally, the cochlear implant surgeon should be aware of this rare organism as a potential cause of otologic infection [7]. Herein, we discuss a challenging case of CI infection with *Mycobacterium abscessus* and review the literature surrounding similar complications.

## 2. Case Report

A 76-year-old female status with a medical history of metastatic papillary thyroid carcinoma, previously treated with thyroidectomy and neck dissection, radioactive iodine, and actively managed on long-term chemotherapy (dabrafenib and trametinib) presented with profound bilateral sensorineural hearing loss and right chronic ear drainage in May 2022. She underwent uncomplicated tympanomastoidectomy at this time at an outside hospital.

**Figure 1:**
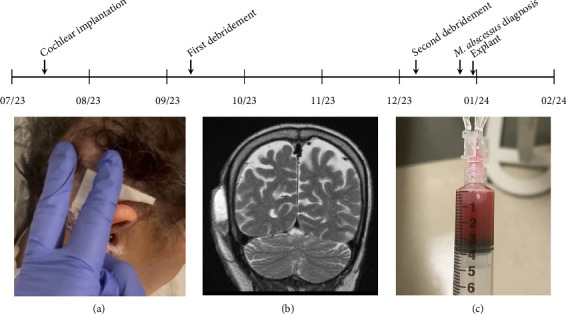
Timeline of pertinent events. Following cochlear implantation, the patient underwent multiple debridements in the operating room, and multiple bedside aspirations. The below images demonstrate representative images from this course. (a) The primary site of postoperative fluctuance was noted at the body of the implant, posterior and superior auricular. (b) A representative MR T2 coronal image of the postoperative subgaleal fluid pocket. (c) The fluid collection was aspirated demonstrating serosanguinous fluid.

She presented for cochlear implant evaluation at our institution and underwent placement of a Cochlear Slim Modiolar Electrode (CI632) in July 2023 (Figure 1). One month after surgery, scant serous drainage was noted from the incision site, and she described continued otalgia. Infection was suspected, and she was prescribed an additional course of cephalexin. Two months after implantation, the patient endorsed symptoms of bleeding and increased drainage from the right postauricular incision site. She was taken to the operating room for right postauricular wound debridement with primary wound closure. Operative findings included granulation tissue, roughly 1.5 cm in length and foreign body reaction at the junction of cochlear implant and antennae exit site from the cochlear implant. Postoperatively, the patient was prescribed cephalexin. In October 2023, one month after debridement and washout, she was noted to have localized granulation tissue at the incision site which was treated with silver nitrate.

In December 2023, there was recurrence of otalgia and fluid collection superior to the incision site, prompting a second operative debridement. Intraoperative bacterial cultures from both debridements had thus far returned negative results. However, fungal and acid-fast cultures had not been sent. At the recommendation of infectious disease, the patient was started on vancomycin and ZOSYN postoperatively and remained admitted for 5 days. On postoperative Day 1, there was noted to be fluctuance at the body of the implant, which was aspirated, demonstrating serosanguinous liquid. She was discharged with a 6-week course of doxycycline and levofloxacin. In late December, the patient's aspirate cultures returned with positive results for *M. abscessus.* She was readmitted for a period of 10 days during which her cochlear implant was explanted. Intraoperatively, there was granulation tissue involving the mastoid cavity. Immediately, postoperatively, she was treated with cefoxitin, azithromycin, omadacycline, and linezolid for 5 days followed by a 5-day course of tedizolid. On postoperative Day 5, she was noted to have fluctuance at the incision site. Bedside aspiration yielded a dark serosanguinous fluid.

At her follow-up visit in late January 2024, the postauricular incision was intact and demonstrated no swelling. The patient reports that she is doing well without otalgia or wound drainage and continues her antibiotic regimen of cefoxitin for 3 months and azithromycin and omadacycline for 6 months. Future treatment plans include a follow-up MRI evaluation to assess the potential for cochlear reimplantation.

## 3. Discussion

In this case report, we demonstrate a rare complication of CI; NTM infection requiring two debridements, two bedside aspirations, and ultimately device explantation. Following device explantation, our patient received a prolonged course of antibiotics. As the literature surrounding NTM cochlear implant infection is sparse, guidance for treatment is not clear. Given that our patient is deaf and the significant impact of a cochlear implant on our patient's quality of life, close monitoring with follow-up imaging will be conducted to assess the patient's progress and possibility of future cochlear reimplantation.

First reported in 1953, the acid-fast Gram-positive rod *M. abscessus* is found commonly in water and soil and has been increasing in prevalence worldwide [9–11]. Though cases of otologic infections with NTM have been recorded, and similarly show increasing prevalence, NTM remains an extremely rare causative pathogen [12–17]. Thorough review of the literature yielded two prior reported cases of *M. abscessus-*infected cochlear implants (Table 1). Given the tendency for this pathogen to form biofilms and its indolent nature, explantation was required in each case. Of the two previously documented cases, only one case ultimately resulted in a successful cochlear reimplantation. The other case reported by Lodhi et al. resulted in explantation (except the intracochlear array) without reimplantation. While we hypothesize this unique complication being facilitated by our patient's history of thyroid cancer and maintenance on chemotherapeutic immune suppression, the two prior reported cases occurred in patients without known immunosuppression.

Disseminated skin and port sit NTM infections occur more frequently in immunocompromised hosts, e.g., patients with AIDS, cancer, previous transplantation, and immunosuppressive drugs [18]. Additionally, the use of medications that suppress T-cell function, such as dabrafenib and trametinib, may place a patient at greater risk for NTM infections as the cell-mediated response to infection is blunted [19, 20]. Given that NTM infections are rare, few reports describe NTM infections within the head and neck, though there are reports involving infection of the temporal bone, sinuses, the middle ear, and the parotid gland [16, 21–23]. The majority of cases were treated with surgery, and all cases were treated with a prolonged course of antibiotics [16, 21–23]. NTM infections can occur in the hip and knee joints with hardware placement, and the majority of these cases result in explanation with prolonged antibiotic treatment [24]. Compared to infection of CI by traditional pathogens such as *S. aureus* and *Pseudomonas* spp. which have explantation rates of 50%–60%, *M. abscessus*-infected CI appear to require a higher rate of explantation as demonstrated in all reported cases [6, 25].

Diagnosis in our case was prolonged and challenging which is not unique among NTM infections. NTM is especially difficult to identify as an infectious source as acid-fast staining can have low sensitivities, between 20% and 70% [26]. Given the difficulty in identifying NTM infections, there are often delays in treatment. Thus, it is critical to consider NTM pathogens in cases with atypical presentation such as indolent, antibiotic infections. Selection of an appropriate treatment strategy depends on various factors including severity of infection, location of infection, patient age, and comorbidities.

Given the challenges in diagnosis of NTM infections, it is important to identify key differences between tuberculosis otomastoiditis (TOM) and NTM-infected CI. Whereas clinically both TOM- and NTM-infected CI can present with otorrhea and hearing loss, TOM is more frequently associated with systemic symptoms such as weight loss, night sweats, and fever [27, 28]. Additionally, though radiologically both TOM and NTM infection may present similarly, NTM infection may also present with granulation tissue [28, 29].

Since NTM infections are rarely reported in the literature and there is a paucity of evidence-based drug regimens for treatment of NTM infection thus, no proven antimicrobial strategy is accepted and long-term therapy is often required [10]. Medical treatment is especially difficult to optimize as *M. abscessus* is prone to develop macrolide resistance. Antibiotic regimen is often focused on the incorporation of additional drugs to help decrease resistance [30]. Commonly used antibiotics include aminoglycosides, beta-lactam antimicrobial agents, macrolides, and fluoroquinolones [31]. The Infectious Diseases Society of America and the American Thoracic Society's guidelines on the diagnosis, treatment, and prevention of nontuberculous mycobacterial diseases state that for serious soft tissue and bone infections, patients should undergo 4–6 months of therapy [10]. When creating a treatment plan for patients using these antimicrobial agents, it is important to consider drug tolerability, adverse reactions, and drug-drug interactions. Additionally, due to the increased resistance antimicrobial agents of NTM species, prolonged treatment with combination antibiotic therapy is often utilized, as in our case [31].

## 4. Conclusion

NTM infections are a rare complication of CI. However, as NTM infections are on the rise globally, it is imperative for the cochlear implant surgeon to consider this pathogen in order to optimize diagnosis and management. We recommend exhaustive culture studies if infection is suspected, early involvement with infectious disease specialists, and explantation upon diagnosis.

## Figures and Tables

**Table 1:** Literature review of cochlear implantation infection by *Mycobacterium abscessus*.

Author	Patient age	Comorbidities	Antibiotic regiment after diagnosis	Outcome
Anderson et al. [8]	7 months	None	Amikacin (10 weeks), cefoxitin (discontinued 3 weeks after initiation due to neutropenia), linezolid, and clarithromycin (35 weeks)	Successful reimplantation
Lodhi et al. [7]	78 years	Chronic deep vein thrombosis	Prolonged treatment with imipenem for 2 months and clarithromycin for 6 months	Explant of device
Current study	76 years	Long-term chemotherapy for metastatic papillary thyroid cancer	Cefoxitin for 3 months and azithromycin and omadacycline for 6 months	Explant of device and planned reimplantation

## Data Availability

The datasets used for the current study are not publicly available due to patient privacy restrictions.
